# Intrathecal Morphine in Major Abdominal and Thoracic Surgery: Observational Study

**DOI:** 10.3390/healthcare13070761

**Published:** 2025-03-28

**Authors:** Silvia González-Santos, Antía Osorio-López, Borja Mugabure-Bujedo, Nuria González-Jorrín, Ane Abad-Motos, Inmaculada Ruiz-Montesinos, Alejandro Herreros-Pomares, Manuel Granell-Gil

**Affiliations:** 1 Department of Anesthesiology, Postoperative Care and Pain Management, Donostia University Hospital, 20014 San Sebastián, Spain; antia.osoriolopez@osakidetza.eus (A.O.-L.); mugabure@yahoo.es (B.M.-B.); nuriagonzalezjo@gmail.com (N.G.-J.); aneabad@hotmail.com (A.A.-M.); 2 Department of Surgery and Radiology and Physical Medicine, Faculty of Medicine and Nursing, University of the Basque Country UPV/EHU, 48940 Leioa, Spain; 3 Department of Gastrointestinal Surgery, Donostia University Hospital, 20014 San Sebastián, Spain; 4 Department of Biotechnology, Universitat Politècnica de València, 46022 Valencia, Spain; 5 Centro de Investigación Biomédica en Red Cáncer, CIBERONC, 28029 Madrid, Spain; 6 Department of Anesthesiology, Postoperative Care and Pain Management, Hospital General Universitario de València, 46014 Valencia, Spain; manuel.granell@uv.es; 7 Department of Surgery, Universitat de València, 46010 Valencia, Spain

**Keywords:** intrathecal morphine, pain in major surgery, postoperative pain, enhanced recovery after surgery programs

## Abstract

**Introduction:** Optimal control of acute postoperative pain after major surgery accelerates the recovery process, shortens hospital stays, and minimizes healthcare costs. Intrathecal morphine is a simple, safe, and reliable regional technique that provides prolonged analgesia, useful in a wide variety of procedures. **Materials and Methods:** A retrospective observational study was conducted on patients who underwent various major abdominal or thoracic surgical procedures and were administered intrathecal morphine between January 2018 and December 2021. The primary objective was to establish the safety of the technique in terms of the incidence of early and late respiratory depression, atelectasis, the need for respiratory support, and the possible association of these complications with the presence of respiratory pathologies such as chronic obstructive pulmonary disease (COPD) or sleep apnea–hypopnea syndrome (SAHS) and obesity or smoking habit. Secondary objectives included recording the consumption of rescue intravenous (IV) morphine in the first postoperative 24 h, the incidence of PONV, and the incidence of late postoperative complications (at 90 days) such as pneumonia, readmission rates, and reoperation rates. Hospital stay and mortality were also recorded. **Results:** A total of 484 patients were included in the study. No patient experienced respiratory depression. Atelectasis occurred in 2.07% of patients. Respiratory support with non-invasive mechanical ventilation (NIMV) or high-flow oxygen therapy (HFOT) was required by 1.86% of patients. In total, 51% of patients required rescue IV morphine (average 6.98 mg), with a rate significantly higher in the thoracic and general surgery groups compared to urological surgery. The incidence of postoperative nausea and vomiting (PONV) was 30.37%. Regarding other secondary objectives, readmissions, reoperations, and mortality rates were significantly higher in patients undergoing urological and thoracic surgery compared to those undergoing general surgery. **Conclusions:** The administration of intrathecal morphine for the control of acute postoperative pain after major surgery can be considered as a safe technique that fits perfectly within the set of measures for a multimodal approach to pain management in major abdominal and thoracic surgery.

## 1. Introduction

Currently, an increasing number of hospitals are implementing Enhanced Recovery after Surgery) (ERAS) programs in numerous surgical processes, with the goal of reducing patient functionality loss, thereby decreasing perioperative morbidity, expediting the recovery process, shortening hospital stays, and minimizing healthcare costs [1]. During the development of these programs, the importance of anesthetic management in achieving the aforementioned outcomes has become evident, creating the need for a multidisciplinary approach and progressively expanding its application to a large number of procedures [1,2]. A crucial element in the success of these programs is the adequate control of perioperative pain to facilitate patient recovery. Therefore, analgesic techniques must be adapted to different surgical approaches and procedures, aiming to address pain in a multimodal manner. This is the objective of the PROSPECT (Procedure Specific Postoperative Pain Management) guidelines, which, based on evidence, aim to optimize pain treatment in various surgical processes [3,4,5,6,7,8,9,10,11]. Currently, regional analgesic techniques are a cornerstone in the multimodal management of surgical patients [12,13]. Numerous regional techniques have been described to date, ranging from classic procedures like epidural analgesia to novel fascial plane blocks. The effectiveness and adverse effects of each technique, depending on the approach and patient characteristics, have been studied by many authors [7,8,12,14,15,16,17].

A classic regional technique, described and used long before the advent of fascial plane blocks, involves the administration of morphine in the intrathecal space. This is a simple technique with a low failure rate and few complications. The previously described regional techniques require a higher level of skill, training, and ultrasound equipment for their implementation [18]. Morphine is a hydrophilic opioid whose use is currently widespread globally. It has multiple administration routes, including the intrathecal route, which has a good profile for the treatment of acute postoperative pain. The use of intrathecal hydrophilic opioids like morphine results in reduced clearance and, therefore, greater persistence of the drug in the cerebrospinal fluid, allowing it to bind to specific receptors located in the gray matter of the spinal cord, providing analgesia over a prolonged period. Additionally, its longer presence in this fluid allows for cephalic migration, which explains the development of side effects, some of which, though infrequent, are severe, such as delayed respiratory depression. This is currently the main reason limiting the use of this technique, as, according to the ASA, ESRA, and ASRA guidelines, it requires the close monitoring of these patients during the initial hours after administration. The dose of intrathecal morphine used has been progressively reduced, demonstrating a similar analgesic efficacy to higher doses, but with a significant reduction in adverse effects [9,19,20,21,22,23].

In our hospital, the use of this regional technique is widespread, creating the need to evaluate, analyze, and share our experience in terms of efficacy and safety. The primary objective of this study was to establish the safety of intrathecal morphine in terms of the incidence of early and late respiratory depression, atelectasis, the need for respiratory support, and the possible association of these complications with the presence of respiratory pathologies such as chronic obstructive pulmonary disease (COPD) or sleep apnea–hypopnea syndrome (SAHS) and obesity or smoking habit. We aimed to determine, if confirmed as a safe technique, the possibility of modifying the standards for the monitored surveillance of these patients for earlier discharge to an inpatient ward following the protocols of ERAS.

Secondary objectives included recording the consumption of rescue intravenous (IV) morphine in the first postoperative 24 h, the incidence of PONV, and the incidence of late postoperative complications (at 90 days) such as pneumonia, readmission rates, and reoperation rates. Hospital stay and mortality were also recorded.

## 2. Material and Methods

After obtaining approval from the local Research Ethics Committee (Date: 22 February 2022, President: Jon Zabaleta Jiménez, Code: GON–MOR–2021–11), we conducted a review of adult patients who underwent major surgery and were administered intrathecal morphine at Donostia University Hospital between January 2018 and December 2021. Data on the study variables’ were collected retrospectively from the clinical history and anesthetic record of each patient included in the study.

The following demographic data of the patients were collected: age (years), sex, weight (kilograms), height (meters), and BMI (kg/m^2^); respiratory comorbidities and smoking habits were also analyzed. Regarding the anesthetic technique used, the doses of intrathecal morphine administered and the presence of associated complications were recorded. The surgical procedure the patient underwent was also coded. Finally, data related to postoperative complications such as respiratory depression, the need for respiratory support, the presence of atelectasis, and nausea and vomiting were collected. Atelectasis was diagnosed based on chest X-ray findings, characterized by lung opacification accompanied by a shift of the mediastinum, hilum, or hemidiaphragm toward the affected area, along with compensatory overinflation of the adjacent non-atelectatic lung. Chest X-rays were routinely performed in patients who underwent major lung resection, presented with hypoxemia (SpO_2_ < 92%), or required ventilatory support.

Intrathecal morphine administration was performed in all cases, with the patient monitored and under conscious sedation, using a 25-gauge needle in the interlaminar space between the second and third or third and fourth lumbar vertebrae (L3 and L4 or L2 and L3). The dose of intrathecal morphine administered was determined based on the responsible professional’s judgment. No specific weight and height criteria were used. Subsequently, patients underwent balanced general anesthesia to proceed with the surgery. There were no changes made due to the administration of intrathecal morphine. The types of surgeries included were as follows: (a) general surgery: colorectal, esophagogastric, hepatic, and pancreatic surgery; (b) urological surgery: cystectomy and nephrectomy; and (c) thoracic surgery: pulmonary resections such as lobectomies, segmentectomies, and atypical resections.

Following surgery, patients were transferred to the postanesthesia care unit (PACU) for monitoring, where the minimum stay was 24 h. The total stay duration depended on the type of surgery and the patient’s clinical progress. Generally, patients who underwent major thoracic surgery, nephrectomies, colorectal surgery, or gastrectomies stayed until the following day. Patients who underwent cystectomy stayed in the PACU for 48 h. Patients received standard non-steroidal anti-inflammatory drugs (NSAIDs) and a rescue morphine protocol, as stipulated by the hospital’s postoperative analgesia guidelines. In this regard, to assess the intensity of pain after surgery, a numeric rating scale (NRS) was used. Patients were periodically asked about their pain, and rescue intravenous (IV) morphine was administered if their score on the scale was higher than 4. Patients who required rescue IV morphine were noted.

**Objectives:** The primary objective was to establish the safety of the technique in terms of the incidence of early and late respiratory depression, defined as bradypnea, with a respiratory rate of fewer than 10 breaths per minute, peripheral oxygen saturation below 90%, and/or signs of drowsiness or deep sedation. The incidence of atelectasis and the need for respiratory support were also analyzed, as well as the possible association of these complications with the presence of respiratory comorbidities, obesity, or smoking habits that may predispose to their development.

The secondary objectives were to record the consumption of rescue intravenous (IV) morphine in the first 24 postoperative hours, the incidence of nausea and vomiting (PONV), and the incidence of late postoperative complications (within 90 days after surgery) such as pneumonia, readmission rates, and reoperation rates. Hospital stays and mortality rates were also recorded.

**Statistical Analysis:** Data are described using the most appropriate statistics for the nature and scale of each variable, as follows: absolute and relative frequencies in percentages and mean and standard deviation (SD) for continuous variables, or median and interquartile range if the data distribution recommended it. To measure the association between categorical variables, the parametric Chi-square test or its non-parametric equivalent, Fisher’s exact test, was used when the parametric test was not applicable. For quantitative variables, normality was verified using the Kolmogorov–Smirnov test, and the ANOVA test was used for comparing means for independent samples, or its non-parametric equivalent (Kruskall–Wallis) when appropriate. A significance level of 0.05 was established. All analyses were performed using the SPSS statistical package (version 29.0.0.0 (241)).

**Literature Review:** A literature search was conducted in the following databases: UpToDate, Medline, Embase, PubMed, and Tripdatabase. The English terms used in the search were intrathecal morphine, intrathecal opioids, pain management for minimally invasive surgery, and perioperative analgesia for minimally invasive surgery.

## 3. Results

**Figure 1:**
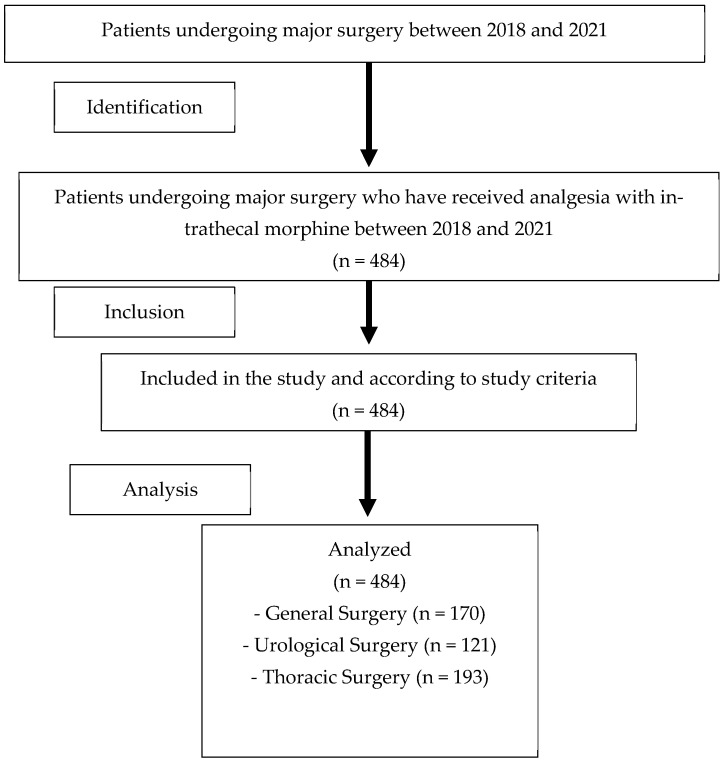
Flow chart.

A total of 484 patients who underwent major surgery at Donostia University Hospital between January 2018 and December 2021 received intrathecal morphine as an analgesic technique (Figure 1. Flow chart).

**Demographics:** The patients had an average age of 65.99 years, were mostly male (60.3%), and had an average height of 166.75 cm and an average weight of 73.26 kg. In total, 37% of the patients were overweight [body mass index (BMI) 25–20] and up to 20% were obese (BMI ≥ 30) (Table 1).

**Preoperative Data:** Preoperative data related to conditions that could increase the risk of respiratory depression and postoperative pulmonary complications were collected. In total, 17.36% of the patients were diagnosed with chronic obstructive pulmonary disease (COPD) or asthma and were receiving chronic bronchodilator treatment, 5.17% had sleep apnea–hypopnea syndrome (SAHS), and 1.4% had both conditions. A total of 0.62% of the patients had interstitial lung diseases. There was also a high percentage of smokers (24.6%) and ex-smokers (25%). The data broken down by surgical specialties are shown in Table 2.

**Types of Surgeries:** The different types of surgeries reviewed are shown in Table 3.

**Technique and Complications:** In the cases reviewed in this study, no adverse incidents were recorded regarding the intrathecal puncture technique. In all cases, it was successfully performed without dosing errors related to morphine. The intrathecal morphine dose administered was based on the mentioned criteria, averaging 180.91 micrograms (mcg) (SD 38.039, with a maximum dose of 400 mcg and a minimum of 100 mcg), significantly higher in the thoracic surgery group (*p* < 0.01).

**Primary Objectives:** No cases of early or late respiratory depression were detected. Regarding respiratory complications, there were ten cases of atelectasis (2.07%) (two in general surgery, one in urological surgery, and seven in thoracic surgery), with no significant differences between surgical groups (*p* = 0.21). Nine patients (1.86%) required respiratory support (zero, five, and four patients, respectively, in the general, urological, and thoracic surgery groups), with these differences being statistically significant (*p* = 0.02) and this need being more frequent in patients undergoing urological or thoracic surgery (Table 4).

In the following diagrams, we show the patients with atelectasis and the need for respiratory support.

**Figure 2:**
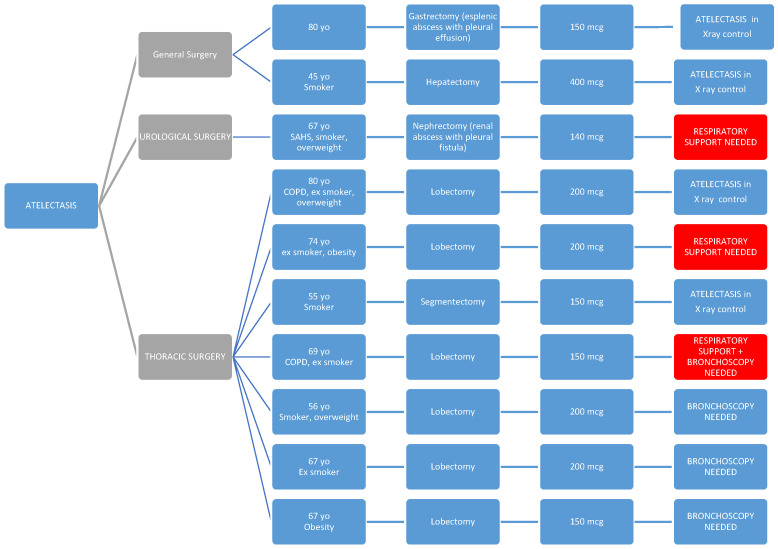
Distribution of patients with postoperative atelectasis across surgical specialties. This diagram illustrates the incidence of atelectasis (n = 10; 2.07%) among patients who received intrathecal morphine, categorized by type of surgery (general, urological, and thoracic). Thoracic surgery patients had the highest number of cases, although the difference was not statistically significant. Patients who also required respiratory support are highlighted in red.

In Figure 2, we see the relationship of patients with atelectasis.

**Figure 3:**
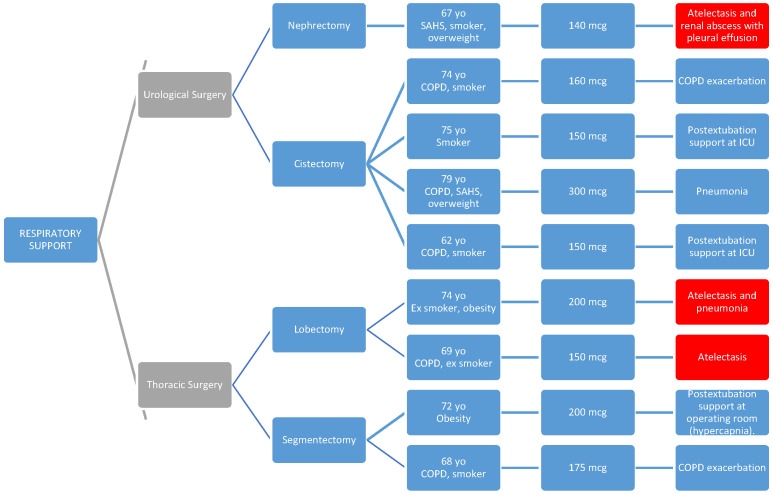
Characteristics of patients requiring postoperative respiratory support. This diagram highlights the subset of patients (n = 9; 1.86%) who required non-invasive mechanical ventilation (NIMV) or high-flow oxygen therapy (HFOT) after surgery. Most of these patients had predisposing factors such as COPD, SAHS, obesity, or smoking history. Patients with concurrent atelectasis are also identified, shown in red. *PACU: post-anesthesia care unit*.

Patients who required respiratory support are highlighted in red, and for this reason, they also appear in Figure 3, where we provide a more detailed reflection of the characteristics of patients who required respiratory support (significantly higher in the urological and thoracic surgery groups).

**Secondary Objectives:** In total, 51.03% (247 patients) required postoperative rescue with intravenous morphine, with an average dose of 6.98 mg (SD 4.984, minimum 1 mg, maximum 30 mg), significantly higher in thoracic surgery patients (*p* < 0.01).

The observed incidence of PONV was 30.37%, with little difference between surgical specialties (32.35%, 28.93%, and 29.53% in the general, urological, and thoracic surgery groups, respectively) (*p* = 0.79). All cases were treated satisfactorily with standard antiemetics (ondansetron and droperidol) at usual doses. Continuing with the description of secondary objectives, the incidence of pneumonia was 3.72% with no differences between surgical groups (*p* = 0.11). The average hospital stay was 9.72 days, longer for patients undergoing urological surgery (16.45 days) and shorter for those undergoing general surgery (10.42 days) and thoracic surgery (4.88 days), with this difference being statistically significant (*p* < 0.01). The readmission rate was 8.06% (*p* = 0.01), and 4.34% of the patients had to be reoperated (*p* = 0.01), with general surgery patients having the lowest incidence of both complications. Six patients died within 90 days post-surgery (1.24%). Four of them had undergone urological surgery, the specialty with the highest mortality incidence (3.31%) (*p* = 0.03), and two were from the thoracic surgery group (Table 5).

## 4. Discussion

The proper control of acute postoperative pain is one of the fundamental pillars of managing patients undergoing major surgery, both open and minimally invasive. With the progressive development of ERAS protocols in abdominal and thoracic surgery through less invasive procedures, the trend is to adjust our anesthetic practice, including regional techniques for pain control [18].

While the use of techniques such as epidural analgesia has been extensively studied in open surgery (e.g., laparotomy, thoracotomy, and cystectomies) [24,25,26], there is less evidence on the management of acute postoperative pain after laparoscopic or minimally invasive surgery [27], the prevalence of which is increasing compared to open surgery, which has been relegated to complex abdominal surgery as far as our data are concerned (as observed in Table 3, both hepatobiliary surgery and cystectomy are performed via laparotomy in all cases, while an open approach in thoracic surgery, for example, is reserved for complex cases requiring extensive pulmonary resections such as pneumonectomies or the presence of intraoperative complications).

Although there is evidence that the approach to pain in such surgeries should consist of a multimodal strategy, with regional techniques as the cornerstone [13], it remains to be determined which regional technique is preferred in these minimally invasive surgeries (e.g., laparoscopies, videothoracoscopies, and robotic surgery). A recent study recommends intrathecal morphine as part of a multimodal analgesic strategy due to its opioid-sparing effect [18,28,29].

The role of epidural analgesia is declining due to its delay in ambulation and discharge home. ERAS Society guidelines no longer recommend the epidural technique in open hepatic resection analgesia [30]. Therefore, in the context of ERAS programs, this technique is being replaced by interfascial regional blocks or surgical site infiltration [4]. Regarding intrathecal morphine, there are conflicting results in the literature. While some consider it as inappropriate as epidural analgesia [4], others view it as an effective, simple method with a low complication rate analgesia [28,31,32]. It has been published that spinal analgesia is the best therapeutic option for postoperative analgesia and opioid reduction in the first 24 h after colorectal surgery, compared to transverse abdominis plane block (TAP), surgical wound catheter, local anesthetic infiltration into incisions, epidural anesthesia, and IV PCA [29].

Likewise, given the opioid-sparing effect of rescue opioids in the postoperative period with the use of intrathecal morphine, as reported by various authors for different types of surgeries [6,28,33,34], it would also perfectly fit within the ERAS protocols, making it ideal for pain management in those patients at a higher risk of respiratory depression due to the use of intravenous opioids [22,30,35,36].

In our study, we observed that 51.03% of the patients (247) required intravenous morphine rescue in the postoperative period, with an average dose of 6.98 mg (SD 4.984 mg, minimum 1 mg, maximum 30 mg). We found that the morphine requirements were significantly lower in the urological surgery group compared to both the general and thoracic surgery groups.

With analgesic efficacy having already been proven in other studies [13,14,15], our aim with this review was to highlight the efficacy of intrathecal morphine in different surgical specialties and also to assess the safety of the technique, especially regarding respiratory depression [5,37,38].

We did not observe any cases of early or late respiratory depression, which have also been reported by other authors using low doses of morphine similar to those used in our study [10]. It is worth noting that the doses of intrathecal morphine administered were low (an average of 180.91 micrograms), which supports previously published findings that doses below 300 micrograms provide adequate analgesia without causing notable side effects [19,39]. Furthermore, this absence of respiratory depression in our study is particularly noteworthy, given that a significant percentage of the patients studied had a medical history of respiratory issues and predisposing conditions to pulmonary complications, which are detailed in Table 3.

In contrast, the meta-analysis by Meylan et al. reported significantly higher rates of respiratory complications and pruritus following intrathecal morphine administration [6]. While that study did not find a linear relationship between the dose administered and the incidence of adverse effects, we believe such a relationship does exist. The discrepancy between our findings and those of Meylan et al. may be attributed to differences in dosing—in several studies included in their meta-analysis, the median intrathecal morphine dose was 500 micrograms—considerably higher than the mean dose used in our cohort.

It is worth noting that a large percentage of COPD patients, smokers, and former smokers underwent thoracic surgery (a predictable observation given the association between smoking, COPD, and lung cancer), with this difference being significant compared to urology and general surgery.

Thus, based on our results and those of other authors regarding the incidence of respiratory depression [1,3,9,16,19,20,29], it can be concluded that the risk with low doses of intrathecal morphine is not greater than that associated with systemic opioid administration analgesia. Therefore, the need for continuous extended monitoring would be unnecessary [1,3,9,16,19,20,29]. Despite a significant difference in the intrathecal morphine doses in the thoracic surgery group compared to the other two, this also does not translate into differences regarding the incidence of respiratory depression, as these are still low doses, as previously mentioned.

As mentioned above, we conducted a review of postoperative respiratory complications such as atelectasis and the need for respiratory support, as well as the presence or absence of preoperative predisposing factors that could increase this risk (such as COPD, OSA, other respiratory diseases, obesity, and smoking or former smoking status).

We want to emphasize that the observation of a higher rate of atelectasis (without this difference being statistically significant) among patients undergoing thoracic surgery may be explained both by the surgery itself and by the fact that, in our center, a chest X-ray is routinely performed on all patients undergoing major lung resection, which is not performed in other specialties. Therefore, in most cases, it may be an incidental finding, as few cases required bronchoscopy or respiratory support.

In all cases, the respiratory issue was detected early and improved with the applied treatment, without requiring reintubation in any of the cases.

As seen in the diagrams, all parients presented one or more of the predisposing preoperative factors such as respiratory pathology (COPD and SAHS), obesity, or smoking habits [40,41].

In four cases, high-flow oxygen therapy (HFOT) or non-invasive mechanical ventilation (NIMV) was used as a transition after orotracheal extubation, and this support could be withdrawn within a few hours.

In terms of the incidence of postoperative pneumonia, no significant differences were found between specialties.

Regarding the observed postoperative PONV rate of 30.37%, it is also in line with what has been published in other series, which may partly be associated with the use of intrathecal morphine [9]. In our experience, this complication was solved in all cases with standard antiemetics (ondansetron and droperidol), although it may be advisable in the future to better tailor the PONV prevention protocol.

No differences were observed between specialties (*p* = 0.79) (32.35% in general surgery; 28.39% in urological surgery; and 29.53% in thoracic surgery), nor were differences observed between subspecialties.

Patients undergoing thoracic surgery required the highest average dose of rescue IV morphine (8.09 mg), followed by general surgery (6.68 mg), with urological surgery patients requiring the least (4.20 mg). These differences suggest variable analgesic adequacy across surgical types, potentially reflecting differing pain intensities or intrathecal morphine dose–response relationships in each context. Importantly, these variations may have clinical implications for tailoring multimodal analgesic regimens based on the surgical procedure. For example, enhanced pain control strategies or supplemental regional blocks may be beneficial in thoracic surgery, where rescue opioid use is higher. Further research could explore whether these differences correlate with functional outcomes, recovery time, or patient satisfaction.

The incidence of readmissions and reinterventions was significantly higher in patients undergoing urological surgery (where mortality was significantly higher) and thoracic surgery compared to those undergoing general surgery. However, hospital stay was longer in the urology and general surgery groups.

The surgical intervention with the highest complication rate was cystectomy, which, in our center, is performed via an open approach (laparotomy) in all cases (during the reviewed period). Out of 95 patients undergoing this procedure, 25 experienced complications, most of them urinary tract infections, with 5 cases requiring readmission to Critical Care Units due to the severity of the pathology. In total, 10 patients required urgent reintervention. A total of 11 patients were readmitted after discharge, and 4 died within 90 days after the intervention. None of the cases involved respiratory complications, but rather complications derived from the surgical technique or the patient’s underlying pathology. This rate does not differ from that described by other authors for this type of surgery [42].

Out of the patients undergoing lung lobectomies, 32 experienced complications, mostly due to persistent air leaks (11 patients) with consequent emphysema and pneumothorax, rates that do not differ from the literature [43]. Out of these 11 patients, 7 needed surgical reintervention and 2 required readmission to Critical Care Units.

Therefore, in patients who are candidates to receive intrathecal morphine as an analgesic technique, it is crucial to carry out a thorough preoperative assessment, defining comorbidities and anticipating possible complications. Additionally, careful intraoperative management along with meticulous postoperative monitoring allows for proper recovery, minimizing the occurrence of complications and enabling early detection in case they occur.

**Study Limitations:** This is an observational, single-center, retrospective study of a large sample of patients; among its limitations, it is a retrospective study with data collection performed afterwards, which may imply information loss. Furthermore, it would be interesting to compare this analgesic technique with intrathecal morphine with other analgesic techniques applicable to the ERAS program; therefore, it might be advisable to conduct a randomized, prospective, multi-center clinical trial in the future to obtain definitive conclusions on the safety and efficacy of intrathecal morphine administration in major surgery.

## 5. Conclusions

The administration of intrathecal morphine at low doses, as used in this study, for the relief of acute postoperative pain in major surgery, including general and digestive surgery, urology, and thoracic surgery, can be considered as a simple, safe, and reliable analgesic technique that would fit perfectly as part of the multimodal analgesic strategy in ERAS protocols for a variety of surgical procedures. With evidence from more than 40 years of low-dose ITM administration, we can conclude that the risk of respiratory depression is not higher than that with systemic opioids, and, therefore, patients could continue their postoperative recovery in hospital rooms without the need for continuous monitoring for an extended period, which could be modified after the evaluation of its clinical safety in a clinical trial in the guidelines of the most important societies, such as ESAIC, ASA, EACTAIC, ESRA, and ASRA.

## Figures and Tables

**Table 1:** Demographics of the patients included in this study.

Variable (*average and standard deviation*)	
**Age (years old)**	65.99 ± 10.96
**Height (cm)**	166.75 ± 9.11
**Weight (kg)**	73.26 ± 14.88
**Males/Females—*n* (%)**	292 (60.3)/192 (39.7)
**ASA physical status I, II, III, IV—*n* (%)**	0 (0), 135 (27.89), 337 (69.63), 12 (2.48)
**General Surgery group**	0 (0), 76 (44.71), 90 (52.94), 4 (2.35)
**Urological Surgery group**	0 (0), 35 (28.93), 81 (66.94), 5 (4.13)
**Thoracic Surgery group**	0 (0), 24 (12.44), 166 (86.01), 3 (1.55)
**BMI (kg/m^2^)—*n* (%)**	
**BMI < 18 (Underweight)**	5 (1)
**BMI 18–24.9 (Normal Weight)**	203 (41.9)
**BMI 25–29.9 (Overweight)**	179 (37)
**BMI 30–24.9 (Obesity class 1)**	79 (16.3)
**BMI 35–29.9 (Obesity class 2)**	16 (3.3)
**BMI > 40 (Morbid Obesity)**	2 (0.4)

ASA, American Society of Anesthesiologists; BMI, body mass index.

**Table 2:** Risk factors for respiratory complications. *p* values refer to the differences in the proportion of patients who had such a condition across the different types of surgeries.

Variable—*n* (%)	*General Surgery*	Urological Surgery	Thoracic Surgery	Total	*p*
**COPD and asthma**	12 (7.06)	24 (19.83)	55 (28.5)	91 (18.8)	<0.01
**SAHS**	5 (2.94)	8 (6.61)	12 (6.22)	25 (5.17)	0.29
**Interstitial lung disease**	0 (0)	1 (0.83)	2 (1.04)	3 (0.62)	0.40
**Smokers**	33 (19.41)	34 (28.10)	52 (26.94)	119 (24.6)	<0.01
**Ex smokers**	16 (9.41)	23 (19.01)	82 (42.49)	121 (25)	<0.01

COPD: chronic obstructive pulmonary disease; SAHS: sleep apnea–hypopnea syndrome.

**Table 3:** Surgical interventions depending on specialty and subspecialty.

	*n* (%)	Open Surgical Approach (%)
***General Surgery***	170 (35.12)	
**Colorectal**	118 (69.42)	17(14.4)
**Esophagogastric**	5 (2.94)	0 (0)
**Hepatobiliary and pancreatic**	32 (18.82)	32 (100)
**Other abdominal surgery**	15 (8.82)	------
***Urological Surgery***	121 (25)	
**Radical cystectomy**	95 (78.51)	95 (100)
**Nephrectomy**	26 (21.49)	2 (7.7)
***Thoracic Surgery***	193 (39.88)	
**Lobectomy**	152 (78.76)	22 (14.47)
**Lung segmentectomy**	24 (12.44)	2 (8.33)
**Wedge resection (also atypical resection)**	17 (8.81)	1 (5.88)

**Table 4:** Incidence of early and late respiratory depression, atelectasis, and need for respiratory support (primary objectives).

Variable (*n*—%)	General Surgery	Urological Surgery	Thoracic Surgery	Total	*p*
**Early respiratory depression**	-	-	-	-	
**Late respiratory depression**	-	-	-	-	
**Atelectasis**	2 (1.18)	1 (0.83)	7 (3.63)	10 (2.07)	0.21
**Respiratory support necessity**	-	5 (4.13)	4 (2.07)	9 (1.86)	0.02

**Table 5:** Postoperative outcomes and analgesic requirements by surgical specialty (secondary objectives).

Variable (*n*—%)	General Surgery	Urological Surgery	Thoracic Surgery	Total	*p*
**IT morphine dose (micrograms)** **(*average*—*SD*)**	178.03 (49.02)	171.32 (36.34)	189.46 (23.69)	180.91 (38.04)	<0.01
**IV morphine dose (milligrams)** **(*average*—*SD*)**	6.68 (4.68)	4.20 (2.23)	8.09 (5.45)	6.98 (4.98)	<0.01
**Nausea and vomiting (*n*—%)**	55 (32.35)	35 (28.93)	57 (29.53)	147 (30.37)	0.79
**Readmission (*n*—%)**	6 (3.53)	12 (9.92)	21 (10.88)	39 (8.06)	0.01
**Pneumonia (*n*—%)**	3 (1.77)	8 (6.61)	7 (3.63)	18 (3.72)	0.11
**Reintervention (*n*—%)**	2 (1.18)	11 (9.09)	8 (4.15)	21 (4.34)	0.01
**Mortality (*n*—%)**	-	4 (3.31)	2 (1.04)	6 (1.24)	0.03
**Hospital stay (*average*—*SD*)**	10.42 (9.49)	16.45 (9.62)	4.88 (3.28)	9.72 (8.93)	<0.01

Readmissions: patients who are re-admitted to the hospital after being discharged home within 90 days after the intervention. Intrathecal (IT); intravenous (IV); IV morphine (mg): the amount of morphine consumption in the first postoperative 24 h.

## Data Availability

Data are contained within the article.
